# Africanized bees extend their distribution in California

**DOI:** 10.1371/journal.pone.0190604

**Published:** 2018-01-18

**Authors:** Wei Lin, Jakob McBroome, Mahwish Rehman, Brian R. Johnson

**Affiliations:** 1 Department of Entomology and Nematology, University of California, Davis, CA, United States of America; 2 Research Officer, Department of Entomology, Agricultural Research Institute, Tarnab, Peshawar, Pakistan; University of Otago, NEW ZEALAND

## Abstract

Africanized honey bees (*Apis mellifera*) arrived in the western hemisphere in the 1950s and quickly spread north reaching California in the 1990s. These bees are highly defensive and somewhat more difficult to manage for commercial purposes than the European honey bees traditionally kept. The arrival of these bees and their potentially replacing European bees over much of the state is thus of great concern. After a 25 year period of little systematic sampling, a recent small scale study found Africanized honey bees in the Bay Area of California, far north of their last recorded distribution. The purpose of the present study was to expand this study by conducting more intensive sampling of bees from across northern California. We found Africanized honey bees as far north as Napa and Sacramento. We also found Africanized bees in all counties south of these counties. Africanized honey bees were particularly abundant in parts of the central valley and Monterey. This work suggests the northern spread of Africanized honey bees may not have stopped. They may still be moving north at a slow rate, although due to the long gaps in sampling it is currently impossible to tell for certain. Future work should routinely monitor the distribution of these bees to distinguish between these two possibilities.

## Introduction

Honey bees are a part of many urban and natural landscapes. In their native range, they are the most populous, large colony bees and they play many important ecological roles [1]. In their introduced range, which includes essentially all land inhabited by human beings with the exception of the two poles, they are a major component of both ecological communities (often as invasive species) and economic systems in which they are used for large scale pollination and food production [2–4]. Many people identify with the honey bee due to its close relationship to human beings and are hence more willing to listen to environmental concerns regarding pollinators at large if they are packaged in the context of keeping honey bees healthy [5–6].

Africanized bees (AHBs) were initially brought to Brazil from South Africa for breeding purposes, but quickly escaped and spread north replacing European honey bees (EHBs) as they moved [7–10]. They arrived in the US in 1990 in Texas and in California in 1994 [11]. The arrival of these bees into California is of concern for two main reasons [12]. First, many parts of California are strongly agricultural and Africanized honey bees can interfere with the use of European honey bees for commercial pollination [11,13]. Second, many regions of California are densely populated and Africanized bees are a public health threat as their defensiveness can lead to serious injury and even death [14].

The purpose of the present study is to document the current distribution of AHBs in California, with emphasis on quantifying their presence in populated areas. Recently, a small scale study found AHBs in the San Francisco Bay Area, which raised much concern in the local popular press [15]. The present study was ongoing before and during that study and uses the same genetic methods. It, however, has a larger scope in terms of number of samples tested across the northern part of the state. It thus can be seen as an extension of Kono and Kohn [15] that both seeks to validate its basic findings and to extend the sampling to more areas. Our goal was to sample from the area of known distribution up to counties in which we could no longer find AHBs. We used a simple genotyping test that makes use of a SNP present in EHBs missing in AHBs [16–17]. As this test was also used in the past, it makes for strong continuity between past measures of AHB distribution and the present measure. We chose to bias our samples to urban areas, although we sampled in rural areas as well, because large numbers of managed EHBs in rural agricultural areas may make detecting the much smaller AHB presence unlikely. Further, urban landscapes are particularly favorable for honey bees in California due to large scale planting of ornamental flowers making food available year-round (something strongly lacking in natural landscapes). Urban landscapes have an abundance of nesting sites as well, while the non-forested landscapes of much of California are not so favorable. Thus, urban landscapes are both where one of the major problems exists (due to the danger of AHBs) and they are also the landscapes most favorable to honey bees (both EHBs and AHBs) suggesting intensive sampling in such areas should be productive for determining the distribution of AHBs.

## Materials and methods

### Collection sites and methods

In urban landscapes, we collected from floral sites separated by at least several kilometers. In rural, and or natural landscapes, we collected bees from wild flowers every 3-5km by driving along roads and highways. We did not collect near farms or from crop plants. Collection locations and number of bees caught at each site are in S1 Table. Bees were collected on flowers by placing a new plastic bag over the flower. Bees flew into the bag, which was then sealed. Bees were then immediately put into 95% ethanol in individual 1.6 ml tubes. Each bag was used once and discarded. For each collection site, the date of collection and the latitude and longitude of the location was recorded. At the end of each collecting day tubes with bees were placed into a refrigerator for storage until DNA extraction.

### Molecular analyses

The flight muscles of bees were used for DNA extraction using the Qiagen DNeasy blood and tissue kit according to the manufacturer’s instructions. To determine if the bee had a mtDNA background of either European or African origin, we followed the protocol of Pinto et al [17]. In short, we amplified a region of the cytochrome b gene with PCR using primers given in Pinto et al [17] and recently used by Kono and Kohn [15]. The PCR product was then digested with BglII at 56°C for 3 hours. European bees have a cleavable site for this restriction nuclease within the amplicon, thus EHBs can be diagnosed by two bands on a standard agarose gel. AHBs exhibit a single band. Any unclear results (faint or double bands and so forth) were thrown out and the test repeated with backup material (tissue or DNA depending on the nature of the PCR failure).

## Results

**Fig 1:**
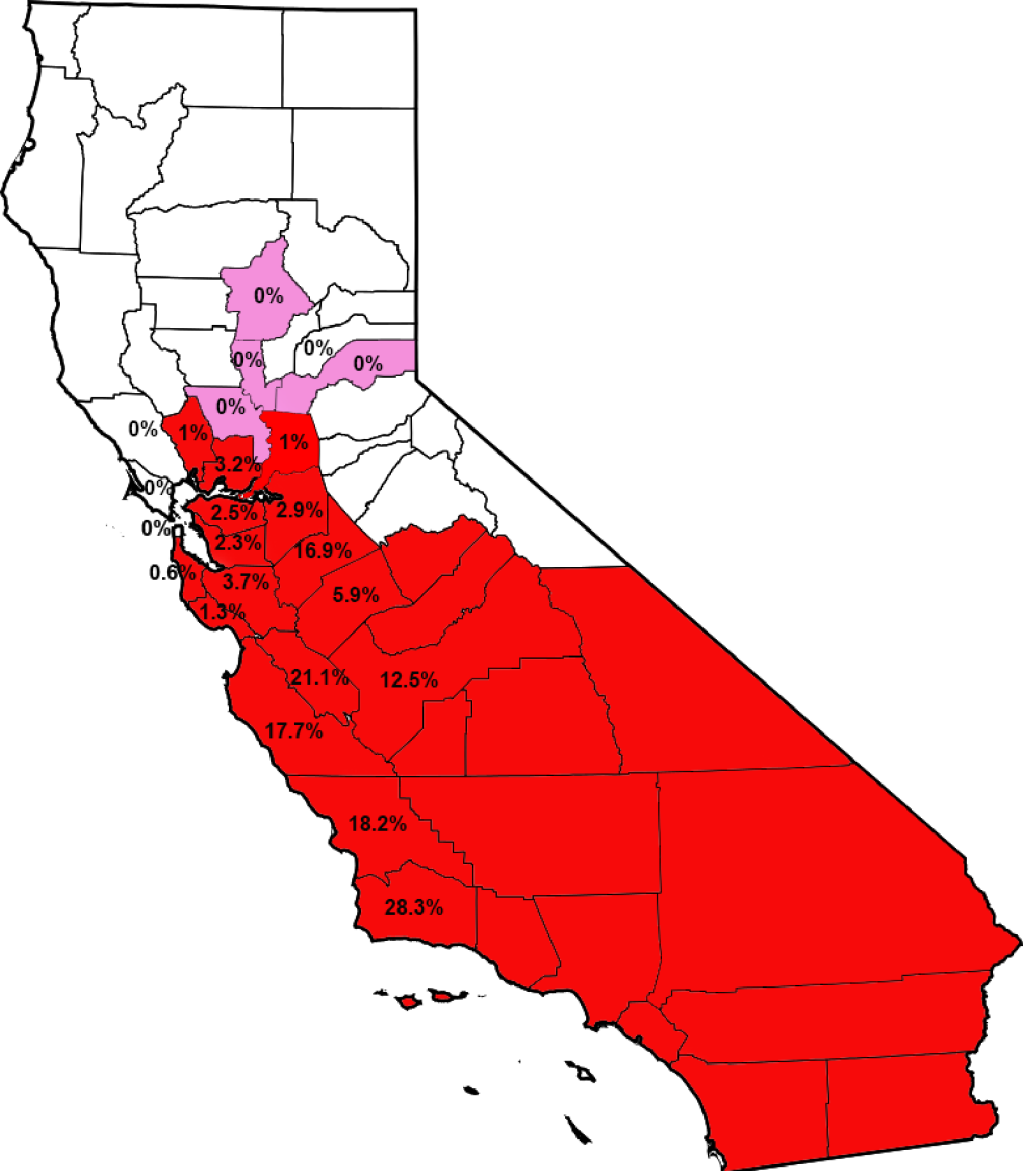
Current distribution of AHBs in California. Percentage of AHBs in each county sampled by the present study only are shown. Counties highlighted in red have been found to be inhabited by AHBs. Counties in pink had no AHBs, but had poor sampling.

We collected and tested 2699 bees from throughout northern California. This spanned the range from where AHBs bee were known to be found (and were common in our dataset) to the region past which we were able to identify AHBs. Fig 1 shows the current distribution of AHBs in California and Table 1 shows the counts of Africanized bees found in each county. The northern counties of Napa and Sacramento now appear to be the northernmost range of AHBs. Neighboring counties along the coast were sampled with some depth but AHBs were not found. Some counties in the central valley and foothills (Yolo and the counties in the Sierra Nevada mountain range below Placer county but above Mariposa) were not sampled. It is likely that AHBs are present in these areas, but future work will have to determine whether this is the case. Counties north of Sacramento (Butte and Shasta, for example) were sampled but AHBs were not found.

**Table 1:** Percentage Africanized and European bees by county.

County	% AHBs	% EHBs	Total
Santa Barbara	28.3	71.7	113
San Benito	21.1	78.9	109
San Luis Obispo	18.2	81.8	154
Monterey	17.7	82.3	496
Stanislaus	16.9	83.1	178
Fresno	12.5	87.5	24
Merced	5.9	94.1	17
Santa Clara	3.7	96.3	191
Solano	3.2	96.8	94
San Joaquin	2.9	97.1	138
Contra Costa	2.5	97.5	277
Alameda	2.3	97.7	43
Santa Cruz	1.7	98.3	177
Napa	1.0	99.0	97
Sacramento	1.0	99.0	96
San Mateo	0.6	99.4	166
Butte	0.0	100.0	29
Marin	0.0	100.0	67
Nevada	0.0	100.0	60
Placer	0.0	100.0	15
San Francisco	0.0	100.0	50
Shasta	0.0	100.0	48
Sonoma	0.0	100.0	44
Sutter	0.0	100.0	10
Yolo	0.0	100.0	6
Total			2699

**Fig 2:**
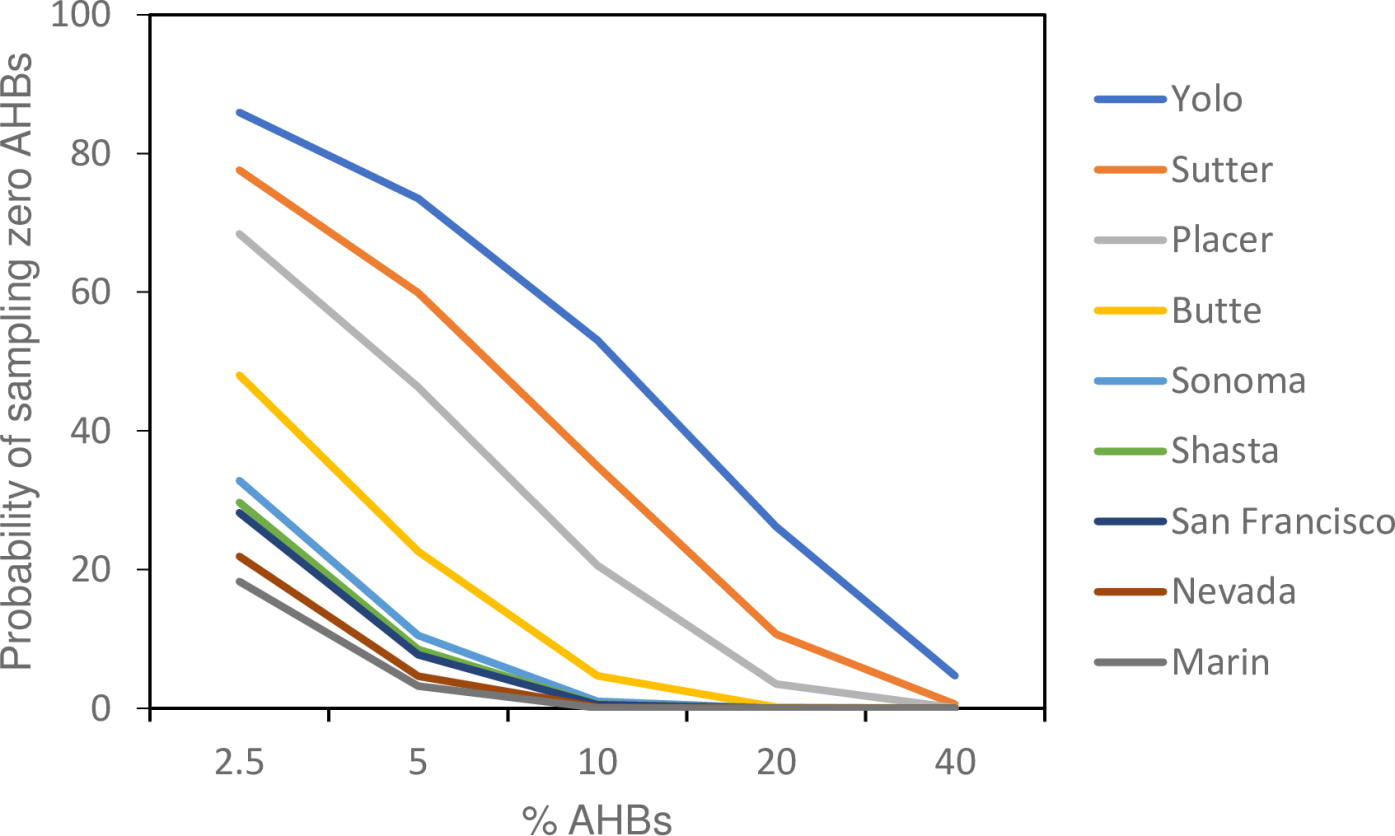
Probability of sampling zero AHBs for all counties sampled without AHB presence using the binomial distribution. For Yolo, for example, there was over 80% chance of sampling zero AHBs assuming AHBs are present at 2.5%. Yolo, Sutter, Placer, and Butte were sampled with insufficient depth to rule out significant AHB presence.

California is a very large state. This, and the difficulty of finding bees in some regions, limited sampling in some counties. Essentially, some counties were poorly sampled and could in principle have AHBs present. Fig 2 explores this issue with a simple analysis using the binomial distribution. Shown is the probability of having sampled no AHBs given their actual presence at various levels in the environment from 2.5 to 40%. For four counties: Yolo, Sutter, Place and Butte, sampling was too limited to conclude that AHBs were not present at even moderately high levels (up to 30–40%). For the other counties in which AHBs were not found, AHBs are likely not present at levels above 5–10%, but could be present at lower frequencies. It is impossible to know the actual probabilities associated with the sampling procedure used, but the idealized situation shown in Fig 2 should be qualitatively correct, meaning roughly speaking AHBs could be present in all of these counties at low levels and could be present at even high levels in the four highlighted counties.

## Discussion

This study was motivated in part by numerous reports to the UC Davis Apiculture program from exterminators in the San Francisco Bay Area complaining of very aggressive bees. Exterminators are often at the vanguard in noticing AHBs in an area, as they are called in to remove unwanted wild or feral bees. Further, it has been nearly 20 years since a large-scale study looked carefully at the distribution of AHBs in California. We found that AHBs are now much further north than they were in the 1990s, being now present in both Napa and Sacramento. AHBs were common in our samples around the Monterey area and in parts of the central valley around Stockton, but they were at very low abundance in Napa and Sacramento. Thus, while the edge of the distribution of AHBs may extend further north than Sacramento, the density of AHBs in these regions is likely quite low. Perhaps the chief take-home message from this study is that the spread of AHBs north may still be occurring at a slow rate. Thus, studies such as the present one should probably be conducted at least once a decade to track their spread.

Kono and Kohn [15] published a recent study using the same methods we used here. They samples over a larger area (up to Oregon), but sampled a smaller number of bees per site and fewer sites. They found AHBs in the East Bay Area (near Oakland and across the bay from San Francisco), which attracted considerable local attention. The present study extends the distribution up to Sacramento and Napa. We also found AHBs over most of the Bay Area, while Kono and Kohn found AHBs in only 2 samples from the East Bay. We thus filled in the distribution of AHBs in the Bay Area and extended it further north.

Interestingly, San Francisco was well sampled in this study and no AHBs were found. This is not due to a lack of forage in this highly urban area. Although San Francisco is the second most densely populated city in the United States, ornamental plants are commonplace on streets and parks and honey bees were quite easy to sample there. We sampled in several large parks with numerous trees (Golden Gate Park, for example,) which provide natural nesting sites. While AHBs may be in San Francisco at low numbers, they are certainly not common and are probably quite rare. Why this is the case is intriguing and could be the focus of future work by local researchers in this area.

Given the extremely rapid spread north of AHBs starting in Brazil in the 1950s (reaching the US in the early 1990s), followed by a very slow rate of spread over the next decade, many researchers suggested that perhaps AHBs had reached their natural northern limit in Central California [8,11]. This was a reasonable assumption given that AHBs are tropical in origin and their spread south into temperate Argentina slowed and stopped when AHBs entered colder climates where presumably EHBs are more competitive [11, 18–19]. The present study suggests that although AHBs have slowed down in their rate of spread, the spread may not be over. California has a Mediterranean climate and is relatively warm throughout the year through most of the central valley and along the coast almost to the Oregon border. It is not clear what the natural northernmost distribution would be in this context. While it is not likely that AHBs will be very common as far north as Redding, it seems reasonable to speculate that they could become common in the Sacramento and Napa areas as these areas do not differ much in climate from regions to the south where AHBs are already common.

We conclude with a discussion of the testing approach used here and by Kono and Kohn (2015). Africanized bees have been shown to be almost exclusively African in their mtDNA haplotype, but up to 20% European in their nuclear DNA [20–21]. European bees outside of the Iberian peninsula, in contrast, are almost exclusive European in their mtDNA and in their nuclear DNA [22–23]. For these reasons, the simple mtDNA test used here has become the standard for tests for Africanized bees in the United States [23–24]. Essentially, a large wave of bees with almost exclusively African mtDNA is spreading into a region with almost exclusively European mtDNA. We can see this in the results of the present study in that in the northern-most samples no African mtDNA was found, while it was abundant in the southern samples.

In general, the present study is best seen as showing that there is a good likelihood that Africanized bees are present in northern California. However, comprehensive studies are now necessary to determine the scope of the problem, which could be small or large. Ultimately, because the present study is only the first step in what must be a series of studies, it is not suitable grounds for policy decisions regarding the large beekeeping industry present in northern California.

## Supporting information

S1 Table Sample information and genotyping results. (XLSX)
